# Effects of a Combined Vitamin D_3_ and Docosahexaenoic Acid Intervention on Cognitive Function and Blood Pyroptotic Biomarkers in Older Adults with Mild Cognitive Impairment: A 12-Month Randomized, Double-Blind, Placebo-Controlled Trial

**DOI:** 10.3390/nu18172777

**Published:** 2026-08-25

**Authors:** Jing Yuan, Ruifang Yang, Hui Chen, Huichao Zhao, Xuan Chen, Ziyan Lou, Xinze Han, Fei Ma

**Affiliations:** 1 Department of Epidemiology and Statistics, School of Public Health, Tianjin Medical University, Tianjin 300070, China; yuanjing5377@tmu.edu.cn (J.Y.); yangruifang2026@163.com (R.Y.); chenxuan88@tmu.edu.cn (X.C.); louziyan@tmu.edu.cn (Z.L.); hanxinze@tmu.edu.cn (X.H.); 2 Department of Obstetric, Gynecologic and Pediatric Nursing, School of Nursing, Tianjin Medical University, Tianjin 300070, China; snchenhui@tmu.edu.cn; 3 Department of Medicine and Humanities, School of Marxism, Tianjin Medical University, Tianjin 300070, China; yelicun@tmu.edu.cn

**Keywords:** mild cognitive impairment, vitamin D_3_, docosahexaenoic acid, NLRP3 inflammasome, pyroptosis, randomized controlled trial

## Abstract

**Background:** This study aimed to assess the effects of a combined vitamin D_3_ (VD_3_) and docosahexaenoic acid (DHA) intervention on cognitive function and blood pyroptotic biomarkers in older adults with mild cognitive impairment (MCI). **Methods:** In total, 360 participants with MCI were recruited, and randomly assigned into four groups: VD_3_ (800 IU/day), DHA (1 g/day), VD_3_+DHA (VD_3_ 800 IU/day+DHA 1 g/day), and placebo. Cognitive function was evaluated by the Wechsler Adult Intelligence Scale-Revised in China (WAIS-RC), and blood biomarkers were measured by ELISA and Western blot at baseline, 6 months and 12 months. Linear mixed-effects models (LMMs) were employed to analyze longitudinal interaction effects on cognitive function and blood biomarkers. Structural equation modeling (SEM) was conducted to evaluate the parallel mediation effects of blood biomarkers on cognitive improvements. **Results:** A total of 355 participants completed the trial. Over the 12-month intervention, all intervention groups demonstrated significant improvements in cognitive function and reductions in five blood biomarkers (IL-1β, IL-18, NLRP3, Gasdermin D (GSDMD), cleaved caspase-1) compared to the placebo group (*p* < 0.05). Compared to each single intervention, the combined VD_3_ and DHA intervention yielded superior cognitive improvements in scores for full-scale IQ (FIQ), performance IQ (PIQ), verbal IQ (VIQ) and several subtests (information, comprehension, digit span, vocabulary, picture completion, block design, and picture arrangement), and significantly reduced five blood pyroptotic biomarkers (all *p* < 0.05). Path analysis revealed that longitudinal GSDMD reduction mediated the improvements in FIQ. **Conclusions:** A 12-month intervention with VD_3_, DHA, or their combination significantly improves cognitive function and mitigates pyroptosis, and the combined intervention showed superior benefits, potentially mediated by downregulating the protein GSDMD.

## 1. Introduction

As global life expectancy rises, dementia, especially Alzheimer’s disease (AD), has become a critical public health priority [1,2]. Mild cognitive impairment (MCI) represents a pivotal transitional stage and a crucial intervention window [3,4]. Despite a potentially bidirectional clinical trajectory, the annual progression rate to dementia among older adults with MCI ranges from 10% to 15%, which is 7–10 times higher than that observed in the non-MCI population [4,5,6]. The trend of cognitive decline is primarily driven by neurodegenerative cascades, where chronic neuroinflammation serves as a key pathogenic factor that exacerbates neuronal damage and synaptic dysfunction [7,8]. Given the current absence of approved pharmacological therapies capable of curing AD or definitively reversing MCI, nutritional care and support should be integrated as a fundamental component of dementia prevention and management strategies [9,10]. Therefore, identifying safe and effective nutritional interventions to delay cognitive decline is an urgent priority.

Among various strategies, nutritional interventions demonstrate significant potential due to their favorable safety profiles [11]. Vitamin D_3_ (VD_3_) and docosahexaenoic acid (DHA) have garnered substantial attention for their neuroprotective properties [12,13]. Low serum 25-hydroxyvitamin D_3_ (25(OH)VD_3_) levels are clinically linked to accelerated cognitive decline [14,15], while DHA serves as a fundamental structural component of neuronal membranes, promoting synaptic plasticity and neuronal survival [16]. Previous evidence also suggests that DHA might modulate the activation of vitamin D receptors (VDRs) and potentially amplify the transcriptional efficacy of VD_3_ [17], indicating that their molecular mechanisms are inherently complementary, and that their combined intervention could surpass the cognitive benefits of single-nutrient interventions. However, previous intervention trials focusing on the effects of single-nutrient interventions have yielded highly heterogeneous results, often confounded by variations in participants’ baseline nutritional status and disease severity [18,19]. Single-nutrient interventions may be insufficient to mitigate complex neurodegenerative pathologies, highlighting the need for multi-target strategies [20]. To date, high-quality evidence regarding the effects of a combined VD_3_ and DHA intervention on cognitive function remains scarce.

The potential of the combined VD_3_ and DHA intervention to improve cognitive function is fundamentally driven by its neuroprotective and complementary anti-inflammatory properties [12]. In the context of neuroinflammation, the innate immune response, particularly the activation of the NLRP3 inflammasome, constitutes a critical mechanism underlying cognitive decline [21,22]. Specifically, overactivation of the NLRP3 inflammasome triggers pyroptosis, a form of programmed cell death characterized by its pro-inflammatory properties, where caspase-1 cleaves Gasdermin D (GSDMD) to execute membrane pore formation and release mature IL-1β and IL-18 [23]. Accumulating evidence indicates that VD_3_ and DHA modulate this pyroptotic pathway through distinct molecular targets, with VD_3_ activating the VDR to directly bind NLRP3 and impede its deubiquitination and assembly [24,25], and DHA signaling via membrane receptors to promote the interaction of the scaffold protein β-arrestin-2 (ARRB2) with NLRP3, thereby inhibiting inflammasome activation [26]. However, whether a combined, long-term intervention with VD_3_ and DHA can exert a superior suppressive effect on the NLRP3-mediated pyroptotic pathway to improve cognitive function remains unexplored. Thus, the present study aims to evaluate the changes in these blood biomarkers, specifically IL-1β, IL-18, NLRP3, GSDMD, and cleaved caspase-1 to explore the potential mechanisms associated with cognitive improvement.

Based on these insights, the combined intervention of VD_3_ and DHA holds immense clinical promise for mitigating cognitive decline. However, clinical evidence from randomized controlled trials (RCTs) investigating the combined effects of VD_3_ and DHA on cognitive function remains virtually non-existent. This 12-month randomized, double-blind, placebo-controlled trial was designed to evaluate the effects of a combined VD_3_ and DHA intervention on improving cognitive function in older adults with MCI by potentially modulating the NLRP3-mediated pyroptotic pathway, thereby providing a robust, mechanism-driven nutritional strategy for mitigating cognitive decline.

## 2. Materials and Methods

### 2.1. Study Population

Participants were recruited from six representative communities in Nankai District, Tianjin, China, utilizing a cluster sampling strategy. Sample size was calculated using PASS software (version 25.0.2; NCSS, LLC, Kaysville, UT, USA). The calculation was conducted based on the changes in cognitive function test scores, utilizing a fixed-effects ANOVA model with primary parameters derived from a previous intervention study involving individuals with MCI [27,28]. Assuming a two-sided significance level (α) of 0.05 and a statistical power (1 − β) of 90%, a minimum sample size of 75 participants per group (total n = 300) was required. To account for a projected attrition rate of 15–20% during the intervention period, the sample size was finally expanded to 90 participants per group, resulting in a total sample of 360 participants.

Participant recruitment was conducted from 5 February to 28 February 2024, based on the following eligibility criteria: (1) aged 65 years or older and (2) maintaining a community-dwelling status (i.e., not residing in or awaiting placement in long-term care facilities). Participants were excluded if they met any of the following conditions: (1) presence of terminal illnesses or severe psychiatric conditions (e.g., major depression, schizophrenia, or bipolar disorder); (2) regular use of vitamin D (VD), fish oil (DHA/EPA), or other nutritional supplements influencing VD metabolism or cognitive function within the three months prior to screening; (3) presence of medical contraindications to VD_3_ or DHA supplementation (e.g., hypercalcemia, active sarcoidosis, or severe coagulation disorders).

**Figure 1:**
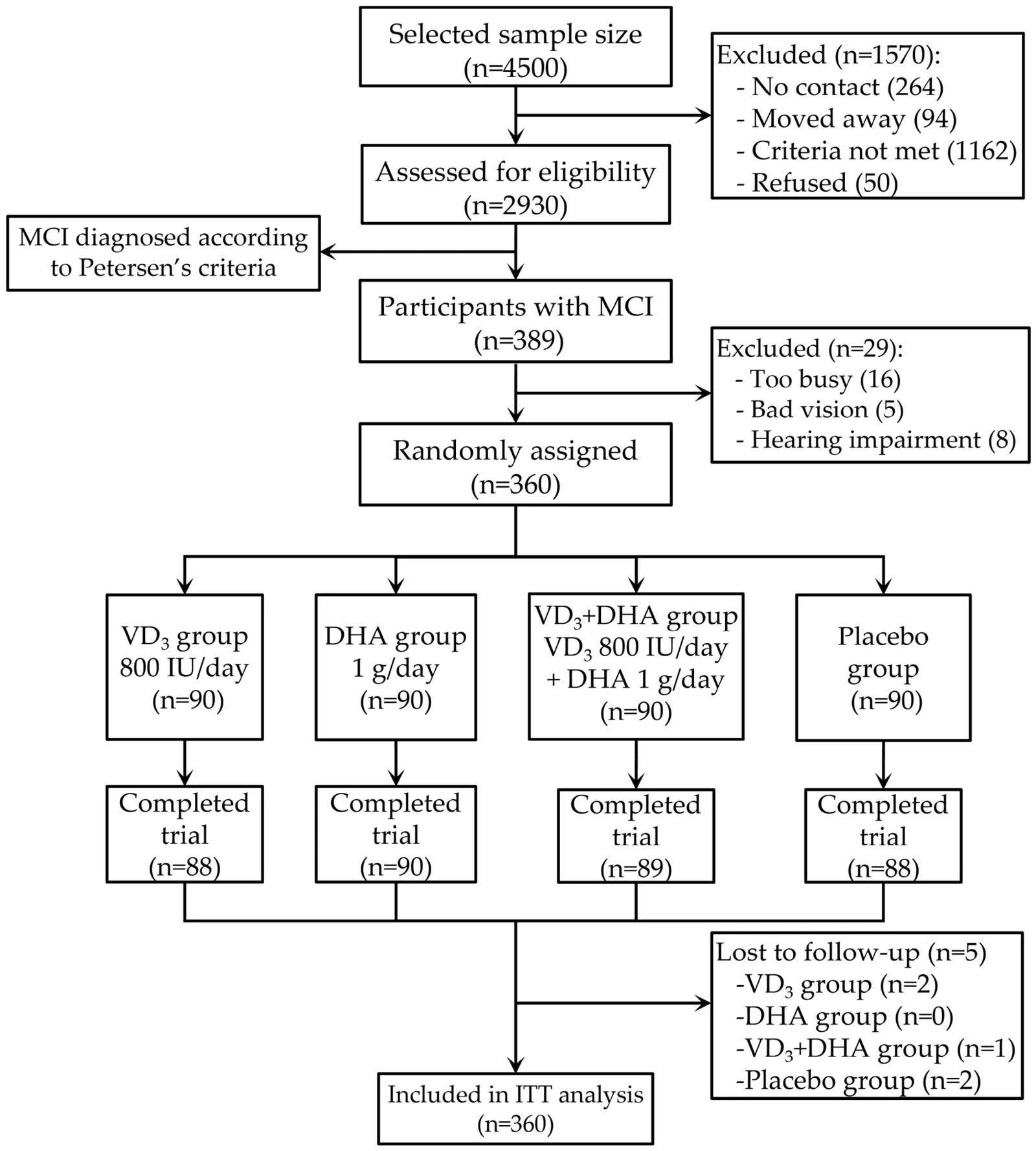
CONSORT flow diagram of the study.

Among the eligible candidates identified through the initial screening, those who were clinically diagnosed with MCI according to modified Petersen’s criteria were ultimately enrolled in the study [29]. The diagnosis of MCI required meeting the following 5 criteria simultaneously: (1) a persistent subjective memory complaint lasting for at least 6 months, independently verified by an informant (family member or physician); (2) an objective memory deficit defined by a score that falls at least 1.5 standard deviations (SD) below the normative mean for age and education on the memory subtask of the Chinese version of the Mini-Mental State Examination (MMSE), and the education-adjusted cut-offs for identifying cognitive impairment were ≤17 for illiterate individuals, ≤20 for those with a primary school education, and ≤24 for those with a secondary school education and above; (3) the preservation of general cognitive performance, demonstrated by global test scores that did not fall below 1.5 SD of age- and education-specific norms; (4) essentially preserved activities of daily living, as measured by an Activities of Daily Living (ADL) Scale score lower than 26; (5) absence of dementia, where the cognitive impairment is insufficient to warrant a diagnosis according to the Diagnostic and Statistical Manual of Mental Disorders-IV (DSM-IV) criteria. Detailed screening and enrollment procedures are illustrated in the CONSORT flow diagram (Figure 1).

The study protocol was approved by the Ethics Committee of Tianjin Medical University (TMUhMEC 2023011) and performed in accordance with the Declaration of Helsinki. Written informed consent was obtained from all participants prior to enrollment. This trial is registered with the Chinese Clinical Trial Registry (ChiCTR2500113843; https://www.chictr.org.cn/showproj.html?proj=288268; retroactively registered due to administrative delays), 3 December 2025.

### 2.2. Assessment of Cognitive Function

Cognitive function was assessed through a battery of standardized neuropsychological tests administered by trained physicians at baseline, 6 months, and 12 months. To maintain the integrity of the double-blind design, all assessors were strictly blinded to participants’ group assignments throughout the study. Cognitive function was evaluated using the Wechsler Adult Intelligence Scale-Revised in China (WAIS-RC) [30]. This comprehensive scale encompasses 11 subtests divided into two main sections: the verbal scale (information, comprehension, arithmetic, similarities, digit span, and vocabulary) and the performance scale (digit symbol, picture completion, block design, picture arrangement, and object assembly). Following each assessment, the obtained raw score of each subtest was initially converted into an age-adjusted scaled score based on the Chinese standardization population norms, with a mean of 10 and a standard deviation of 3 [30]. Subsequently, the sums of the scaled scores from the 6 verbal subtests, the 5 performance subtests, and all 11 subtests were mapped to their respective Chinese standardization conversion tables to derive the verbal intelligence quotient (VIQ), performance intelligence quotient (PIQ), and full-scale intelligence quotient (FIQ).

### 2.3. Laboratory Assay

Fasting venous blood samples were collected at baseline, 6 months, and 12 months after an overnight fast (10–12 h). Samples were centrifuged at 3000 rpm for 10 min, and the serum was stored at −80 °C until analysis. Total cholesterol (TC) and triglycerides (TGs) were measured using an automatic biochemistry analyzer (Hitachi 7180, Hitachi High-Technologies Corporation, Hitachinaka, Japan) with commercial kits. High-density lipoprotein cholesterol (HDL-C) was determined using a Hitachi 911 auto-analyzer. Low-density lipoprotein cholesterol (LDL-C) was estimated using the Friedewald equation: LDL-C = TC − HDL-C − (TG/5) [31]. The levels of serum 25(OH)VD_3_ and 1,25-dihydroxy vitamin D_3_ (1,25(OH)_2_VD_3_) and erythrocyte DHA concentration were quantified using high-performance liquid chromatography–tandem mass spectrometry (HPLC-MS/MS) on an API4000 system (AB SCIEX, Carlsbad, CA, USA). Serum levels of pro-inflammatory cytokines (IL-1β and IL-18) and amyloid markers (Aβ_40_ and Aβ_42_) were quantified using enzyme-linked immunosorbent assay (ELISA), with the Aβ_42/40_ ratio calculated as a marker of amyloid burden. Protein expression levels of amyloid precursor protein (APP), presenilin-1 (PS1), and presenilin-2 (PS2), as well as key NLRP3 inflammasome and pyroptosis pathway proteins, including NLRP3, GSDMD, and cleaved caspase-1, were evaluated via Western blot (WB). The experimenters were strictly blinded to the intervention group allocations during the execution of the assays and the subsequent data quantification.

### 2.4. Intervention, Randomization, and Blinding

During the 12-month intervention period, participants were randomly assigned to one of four groups in a 2 × 2 factorial design: (1) the VD_3_ group, receiving 800 IU/day of cholecalciferol (administered as two 400 IU capsules) plus one DHA placebo; (2) the DHA group, receiving 1 g/day of DHA (one capsule) plus two VD_3_ placebos; (3) the VD_3_+DHA group, receiving both 800 IU/day of cholecalciferol (two capsules) and 1 g/day of DHA (one capsule); (4) the placebo group, receiving dual placebos (two VD_3_ placebos and one DHA placebo). To ensure a rigorous double-blind design, all active supplements and their corresponding placebos, which contained corn oil, were manufactured by the supplier to be identical in appearance, color, shape, size, packaging, and odor. All participants were advised to adhere to their prescribed capsule regimen on a daily basis, with a preference for administration post-meal to enhance nutrition adsorption. Compliance with this regimen was closely monitored through biweekly pill counts.

### 2.5. Follow-Up

Following the comprehensive baseline evaluations, follow-up visits were conducted at 6 and 12 months. These visits focused on replicating the core outcome assessments of cognitive function and blood biomarkers to evaluate the intervention’s effects. Additionally, adverse events and trial compliance were monitored at each interval.

### 2.6. Outcomes

Primary Outcome: The primary outcome was the change in comprehensive cognitive function, specifically evaluated via the FIQ, VIQ, and PIQ, from baseline to month 12.

Secondary Outcomes: Secondary outcomes included changes in nutritional biomarkers (25(OH)VD_3_, 1,25(OH)_2_VD_3_, and erythrocyte DHA), cognitive pathology markers (Aβ_42/40_, APP, PS1, and PS2), and mechanistic profiles associated with the NLRP3/pyroptosis signaling axis and systemic inflammation (NLRP3, GSDMD, cleaved caspase-1, IL-1β, and IL-18) assessed at baseline and month 12.

### 2.7. Statistical Analysis

All analyses were conducted using R version 4.5.2 (R Foundation for Statistical Computing, Vienna, Austria). Statistical differences were examined with *χ*^2^ tests or Fisher’s exact test for categorical variables and one-way ANOVA for continuous variables, with post hoc comparison using the Bonferroni test for multiple comparisons. Linear mixed-effects models (LMMs) were employed to estimate longitudinal interaction effects on cognitive function and blood biomarkers, expressed as *β* coefficients with 95% confidence intervals (CIs). A random intercept for each participant was included to account for within-subject correlations across repeated measures in these models. Specifically, two-way interactions (Group × Time) were used to evaluate the effects of each intervention group compared to the placebo group, while the combined intervention (VD_3_+DHA) group was set as the reference to evaluate the effects of the combined intervention compared to each single nutritional intervention over the 12-month intervention. Two distinct models were developed to assess longitudinal changes in the intervention groups at 6-month (Model 1) and 12-month (Model 2) follow-ups. All models were adjusted for age, sex, education level, BMI, smoking status, alcohol consumption, family history, medical history, and baseline value of the respective outcome. Statistical analyses were performed using the intention-to-treat (ITT) principle, including all randomized participants. Missing data were accommodated within the LMMs using maximum likelihood estimation under the missing at random (MAR) assumption. A two-sided *p* < 0.05 was considered to be statistically significant.

To further explore the mechanisms behind these superior longitudinal effects, path analysis via structural equation modeling (SEM) was performed by comparing the VD_3_+DHA group against the other three study groups. Parallel mediation models included the primary cognitive outcomes (FIQ, VIQ, and PIQ) and the five blood pyroptotic biomarkers that exhibited significant Group × Time interaction effects in the longitudinal LMM analysis over the 12-month intervention. The longitudinal changes in these biomarkers served as parallel mediators, whose total, direct, and indirect paths to changes in cognitive function were estimated using the bias-corrected bootstrap method with 1000 resamples, adjusting for age, sex, education level, BMI, smoking status, alcohol consumption, family history, medical history, and the baseline value of the respective outcome. The effect size of the mediation was explicitly defined and calculated as the ratio of the indirect effect to the total effect. Model fit was evaluated using the Comparative Fit Index (CFI), Tucker–Lewis Index (TLI), Root Mean Square Error of Approximation (RMSEA), and Standardized Root Mean Square Residual (SRMR). Acceptable fit was defined a priori as CFI and TLI ≥ 0.90, and SRMR ≤ 0.08. Models exhibiting excellent CFI (>0.95) and SRMR (<0.08) were considered to have adequate overall fit, even if RMSEA marginally exceeded the 0.08 threshold [32], as small degrees of freedom (*df* < 50) can artificially inflate RMSEA [33].

## 3. Results

### 3.1. Characteristics of Participants

A total of 360 participants were randomly assigned to four groups (n = 90 each). Following the 12-month intervention period, 355 subjects successfully completed the study, with 88 remaining in the VD_3_ group, 89 in the DHA group, 89 in the VD_3_+DHA group, and 89 in the placebo group. Five subjects withdrew during the follow-up period and statistical analysis indicated no significant differences in baseline demographic characteristics between the subjects who withdrew and those who completed the trial (*p* > 0.05). The detailed flow diagram of enrollment, screening, and allocation is illustrated in Figure 1. During the 12-month intervention, the mean medication adherence rates were 93.2% ± 5.1% in the placebo group, 92.6% ± 5.6% in the VD_3_ group, 93.8% ± 4.8% in the DHA group, and 91.9% ± 6.0% in the VD_3_+DHA group. There were no statistically significant differences in adherence among the four groups (*p* > 0.05). No adverse events were reported in any group throughout the 12-month intervention.

At baseline, no significant differences were observed among the four groups in terms of core demographic characteristics, including age, sex, and education levels (categorized as primary school or below, secondary school, and university or above) (all *p* > 0.05). Similarly, lifestyle factors, medical history, and cognitive and functional scores, such as MMSE and ADL, were balanced across all groups (all *p* > 0.05). Furthermore, biochemical measures such as serum 25(OH)VD_3_ and 1,25(OH)_2_VD_3_, and erythrocyte DHA levels were comparable across all groups at baseline (*p* > 0.05). These baseline characteristics are detailed in Table 1.

### 3.2. Effects of Interventions on Cognitive Function in MCI

Table 2 summarizes the cognitive function scores at baseline, 6 months, and 12 months. At baseline, all cognitive indices, including IQ and individual subtest scores, were comparable across the four study groups (all *p* > 0.05), demonstrating the efficacy of the randomization process. As the intervention progressed, statistically significant inter-group differences emerged at the 6-month and 12-month follow-ups (*p* < 0.05). Specifically, over the 12-month intervention, significant differences among the four groups were observed in scores for FIQ, VIQ, and PIQ, as well as the following subtests: information, digit span, vocabulary, arithmetic, comprehension, similarities, picture completion, block design, digit symbol, and picture arrangement (*p* < 0.05).

As shown in Table 2, LMMs were employed to assess the interaction effects (Group × Time) over the longitudinal follow-up. Over the 12-month intervention, all three intervention groups demonstrated statistically significant cognitive improvements compared with the placebo group (all *p* < 0.05). Specifically, the VD_3_ group exhibited improvements in scores for FIQ, VIQ, and the subtests of information, comprehension, arithmetic, digit span, vocabulary, and block design; the DHA group displayed improvements in VIQ and the specific subtests of information, similarities, digit span, and picture completion; and the VD_3_+DHA group demonstrated the most comprehensive improvements, encompassing FIQ, VIQ, PIQ, and the subtests of information, comprehension, arithmetic, digit span, vocabulary, digit symbol, block design, and picture arrangement. To further assess the effects of the combined intervention, the VD_3_+DHA group was set as the reference in LMMs. The results demonstrated that, compared to the combined intervention, both the single VD_3_ group and the single DHA group exhibited significantly less improvement in scores for FIQ, VIQ, PIQ, information, comprehension, digit span, vocabulary, picture completion, block design, and picture arrangement over the 12-month intervention (all *p* < 0.05). Additionally, for the arithmetic subtest, the single DHA group showed significantly less improvement compared to the combined group (*p* < 0.05), whereas no significant difference was observed between the single VD_3_ group and the combined group (*p* > 0.05).

### 3.3. Effects of Interventions on Blood Biomarkers in MCI

The study monitored various blood biomarkers to evaluate the effectiveness of the nutritional intervention. As shown in Table 3, serum levels of 25(OH)VD_3_ and 1,25(OH)_2_VD_3_ and erythrocyte DHA levels were comparable across the four groups at baseline (*p* > 0.05), whereas these biomarkers significantly increased in their respective intervention groups at the 6-month and 12-month follow-ups (*p* < 0.05). Longitudinal LMM analysis over the 12-month intervention confirmed that serum 25(OH)VD_3_ and 1,25(OH)_2_VD_3_ levels in the VD_3_+DHA group and VD_3_ group were significantly higher than those in the placebo group (*p* < 0.05). Similarly, erythrocyte DHA concentration in the VD_3_+DHA group and DHA group was significantly higher compared to the placebo group (*p* < 0.05). Collectively, the clinical results indicate that the interventions effectively modulated nutritional status in participants.

Regarding AD-related pathological biomarkers, the Aβ_42/40_ ratio and the levels of APP, PS1, and PS2 showed no significant differences among the four groups at baseline or at the 6-month and 12-month follow-ups, and LMMs further demonstrated that none of the intervention groups exhibited statistically significant differences compared with the placebo group over the entire 12-month period (all *p* > 0.05).

To explore the underlying mechanisms, a panel of five key blood biomarkers related to NLRP3-mediated neuroinflammation (IL-1β, IL-18, NLRP3, GSDMD, and cleaved caspase-1) was measured. As shown in Table 3, the baseline levels of these five biomarkers were comparable across the four groups (*p* > 0.05), whereas significant differences emerged at the 6-month and 12-month follow-ups (*p* < 0.05). Based on the analysis of two-way interactions (Group × Time), compared to the placebo group, significant reductions in IL-1β, NLRP3, GSDMD, and cleaved caspase-1 were observed in both the VD_3_ group and DHA group (*p* < 0.05), and significant reductions were observed in IL-1β, IL-18, NLRP3, GSDMD, and cleaved caspase-1 in the VD_3_+DHA group (*p* < 0.05). Moreover, to further assess the effects of the combined intervention on pyroptosis biomarkers, the VD_3_+DHA group was set as the reference in the LMMs. The results demonstrated that, compared to the combined intervention, both the VD_3_ group and the DHA group exhibited significantly less reduction in the levels of IL-1β, IL-18, NLRP3, GSDMD, and cleaved caspase-1 over the 12-month intervention (all *p* < 0.05).

### 3.4. Parallel Mediation of the Combined Intervention’s Effects on Cognitive Function via Pyroptotic Pathways

SEM was conducted to evaluate parallel mediation pathways, using the longitudinal 12-month changes in IL-1β, IL-18, NLRP3, GSDMD and cleaved caspase-1 as mediators to assess the mediation effects on improvements in scores for FIQ, VIQ, and PIQ (Table 4). The mediation models for all three cognitive outcomes demonstrated adequate to excellent global fit (e.g., all CFI > 0.94, SRMR ≈ 0.02), with detailed fit indices summarized in Supplementary Table S1. Parallel mediation analysis revealed that statistically significant total indirect effects were exclusively identified for FIQ (*p* < 0.05), while no significant total indirect effects were detected for VIQ and PIQ, information, or comprehension (*p* > 0.05). Specifically, for FIQ, the significant total indirect effect accounted for 10.6% of the total effect of the intervention (*p* < 0.05), which was uniquely driven by the independent indirect pathway via GSDMD (*p* < 0.05, effect size: 7.7%).

## 4. Discussion

In this 12-month randomized, double-blind, placebo-controlled trial, the combined VD_3_ (800 IU/day) and DHA (1 g/day) intervention significantly improved cognitive function (FIQ, VIQ, PIQ, and the specific subtests of information, comprehension, arithmetic, digit span, vocabulary, digit symbol, block design and picture arrangement) compared to the placebo group, and demonstrated significant superior improvements in scores for FIQ, PIQ, information, comprehension, and picture completion compared to each single nutritional intervention. Furthermore, this combined VD_3_ and DHA intervention significantly reduced blood pyroptotic biomarkers (IL-1β, IL-18, NLRP3, GSDMD, and cleaved caspase-1) compared to the placebo group, and demonstrated significant reductions in IL-1β, IL-18, and GSDMD compared to each single nutritional intervention. Parallel mediation analysis demonstrated that the longitudinal reductions in GSDMD exerted a positive mediation effect on the improvement in FIQ.

Our findings indicated that the combined VD_3_ and DHA intervention yields superior cognitive improvements compared to each single intervention, particularly in scores for FIQ, PIQ, information, comprehension, and picture completion. The potential mechanisms underlying this enhanced effect may involve the individual neuroprotective roles of these nutrients. The primary symptoms of cognitive decline caused by AD involve memory loss and a decreased ability to think, analyze, and judge [2,34]. Consistent with our findings, previous RCTs evaluating individual VD3 supplementation in MCI and AD patients have reported enhanced protective effects on executive function and episodic memory, significantly delaying cognitive decline when initiated in the MCI stage [35,36]. VD_3_ can bind to the VDR and exert transcriptional regulatory effects by directly inhibiting pro-inflammatory signaling pathways, reducing amyloid beta (Aβ)-induced neuroinflammation, and maintaining neuronal integrity [37,38]. Similarly, previous RCTs have indicated that supplementing with DHA can significantly enhance spatial learning and attention in patients with MCI [39,40], and maintaining high plasma levels of DHA may also lower the risk of MCI patients developing dementia [41]. Unlike the transcriptional regulatory pathway of VD_3_, DHA helps build a stable defense barrier for the nervous system by promoting the resolution of inflammation and neutralizing neurotoxicity [42]. Therefore, the greater degree of cognitive improvement observed in the combined intervention group offers supporting clues regarding the complementary mechanisms of VD_3_ and DHA, which indicates that a combined strategy may provide broader benefits than single interventions, thereby potentially validating earlier animal-based hypotheses in human clinical practice [12].

In this trial, the 12-month combined intervention significantly reduced blood pyroptotic biomarkers related to NLRP3-mediated neuroinflammation, particularly IL-1β, IL-18, and GSDMD, showing superior effects compared to single interventions. This partial suppression by each single nutritional intervention is inadequate to halt neuroinflammation because cellular pyroptosis functions as a tightly connected cascading pathway [22,43]. Once the NLRP3 inflammasome is activated, it inevitably initiates a proteolytic cascade where caspase-1 cleaves GSDMD to execute membrane pore formation, a defining feature of cellular pyroptosis [44,45]. Recent studies demonstrate that VD_3_ and DHA modulate this pyroptotic process through distinct yet highly specific molecular targets, with VD_3_ signaling via the VDR to impede complex assembly [46], and DHA acting through GPR40/120 and ARRB2 to halt inflammasome activation [47]. By simultaneously targeting these dual molecular targets, ranging from upstream transcriptional regulation to downstream inflammatory resolution, the combined intervention provides a unique dual-target containment that effectively prevents cellular pyroptosis, thereby halting the maturation and massive secretion of potent pro-inflammatory cytokines, particularly IL-1β and IL-18 [48]. Crucially, our parallel mediation analysis bridges this biochemical blockade with clinical outcomes, revealing that the longitudinal reduction in GSDMD acts as a key biological mediator driving cognitive improvements. Specifically, this reduction in GSDMD exerted a robust positive mediation effect on FIQ. As the ultimate executioner of pyroptosis, the downregulation of GSDMD not only preserves neuronal membrane integrity but also fundamentally restricts the extracellular leakage of neurotoxic cytokines and danger-associated molecular patterns (DAMPs) [49,50]. This broad suppression of neuroinflammation closely aligns with the global cognitive improvements captured by the FIQ score, highlighting the potential of modulating the pyroptotic pathway for the early prevention and management of MCI.

Furthermore, the present study demonstrated no statistically significant association between the combined intervention and the Aβ_42/40_ ratio or Aβ-related proteins (APP, PS1, PS2). These findings suggest that within the 12-month intervention period, the superior cognitive improvements of the combined VD_3_ and DHA intervention are highly consistent with the modulation of the NLRP3-mediated pyroptotic pathway, rather than direct alteration of Aβ processing or clearance [8,51]. This absence of Aβ modifications likely reflects a distinct mechanism priority and restricted time window, as the dynamic inflammatory cascade responds acutely to nutritional modulation, whereas clearing established amyloid pathology is a time-intensive process requiring a significantly longer intervention [52,53,54]. Although previous clinical trials on DHA or VD_3_ supplementation reported notable modifications in Aβ-related biomarkers, such discrepancies are primarily driven by methodological differences in intervention durations, dosages, and patient clinical stages [55,56]. The present findings suggest that during the transitional MCI stage, the highly dynamic NLRP3-mediated inflammatory cascade represents a far more sensitive and modifiable therapeutic target than classic amyloid pathology, serving as the predominant driver through which macro-nutritional intervention exerts its cognitive benefits [57,58]. However, these results should be interpreted with caution, as potential confounding factors such as baseline dietary habits, physical exercise levels, and APOE genotype were not fully controlled for. Future studies should incorporate neuroimaging techniques, such as positron emission tomography (PET), to directly verify central neuroinflammatory responses and Aβ burden.

The primary strengths of the present study are as follows. First, a rigorous 12-month, double-blind, placebo-controlled 2 × 2 factorial RCT was conducted to provide clinical evidence for the superior effect of a combined VD_3_ and DHA intervention in MCI patients. Second, the successful implementation of randomization ensured well-balanced baseline characteristics across all four study groups, thereby enhancing the statistical validity of subsequent multi-domain cognitive assessments and blood biomarker evaluations. Third, not limited to the classical Aβ hypothesis, the present study revealed that the combined intervention suppresses the NLRP3-mediated pyroptotic pathway through complementary molecular targets, particularly identifying GSDMD as a potential key mediator associated with cognitive improvements. However, several limitations have to be noted. First, the participants were recruited from a single center, which may limit the generalizability of our findings. Second, the control for potential confounders, including dietary habits, physical activity, and genetic factors like the APOE genotype, was not exhaustive. Finally, although blood biomarkers often reflect systemic inflammation, they do not provide a direct measure of central nervous system pathophysiology. Future multi-center trials with larger, diverse populations are necessary to validate our findings.

## 5. Conclusions

In conclusion, interventions with VD_3_ (800 IU/day), DHA (1 g/day), or their combination for 12 months can significantly improve cognitive function and mitigate systemic pyroptosis in older adults with MCI, and the combined intervention showed superior benefits compared to each single nutritional intervention. The cognitive improvements from the combined VD3 and DHA intervention are strongly associated with the downregulation of the NLRP3-mediated pyroptotic pathway, suggesting the potential involvement of the pyroptosis-executing protein GSDMD. These findings highlight the potential of this combined supplementation as a safe, accessible, and mechanism-driven nutritional strategy for early intervention in cognitive decline. Further large-scale, randomized, double-blind, placebo-controlled trials with extended follow-up are warranted to validate these findings.

## Figures and Tables

**Table 1:** Baseline characteristics of the study population.

	VD_3_ (*n* = 90)	DHA (*n* = 90)	VD_3_+DHA (*n* = 90)	Placebo (*n* = 90)	*p*
Demography					
Age (years)	69.31 ± 5.79	69.32 ± 6.26	68.62 ± 4.45	69.57 ± 6.12	0.712
Males, *n* (%)	37 (41.1%)	44 (48.9%)	44 (48.9%)	42 (46.7%)	0.691
Education, *n* (%)					0.313
Primary school or below	15 (16.7%)	10 (11.1%)	20 (22.2%)	15 (16.7%)	
Secondary school	64 (71.1%)	72 (80.0%)	56 (62.2%)	65 (72.2%)	
University or above	11 (12.2%)	8 (8.9%)	14 (15.6%)	10 (11.1%)	
Lifestyle					
Smoking (never), *n* (%)	7 (7.8%)	7 (7.8%)	8 (8.9%)	7 (7.8%)	0.992
Alcohol (never), *n* (%)	2 (2.2%)	3 (3.3%)	2 (2.2%)	3 (3.3%)	0.998
BMI, kg/m^2^	25.59 ± 4.31	24.63 ± 4.90	24.60 ± 4.11	24.85 ± 3.75	0.373
Medical history					
Past history (never), *n* (%)	39 (43.3%)	50 (55.6%)	45 (50.0%)	47 (52.2%)	0.411
Family history (never), *n* (%)	15 (16.7%)	29 (32.2%)	23 (25.6%)	29 (32.2%)	0.058
Biochemical measures					
Glucose (mmol/L)	5.67 ± 1.38	5.78 ± 1.40	5.81 ± 1.96	5.86 ± 1.73	0.888
TC (mmol/L)	4.82 ± 1.09	4.85 ± 1.06	4.86 ± 1.11	4.84 ± 1.07	0.993
TG (mmol/L)	1.54 ± 0.85	1.55 ± 0.78	1.71 ± 0.86	1.60 ± 0.77	0.466
HDL-C (mmol/L)	1.58 ± 0.31	1.53 ± 0.24	1.54 ± 0.24	1.50 ± 0.28	0.297
LDL-C (mmol/L)	2.25 ± 0.82	2.47 ± 1.60	2.16 ± 0.92	2.36 ± 1.22	0.326
25(OH)VD_3_	19.43 ± 3.38	19.97 ± 3.42	19.76 ± 3.38	19.54 ± 2.64	0.678
1,25(OH)_2_VD_3_	30.21 ± 5.95	31.10 ± 4.86	29.85 ± 4.34	29.37 ± 5.14	0.138
Erythrocyte DHA	3.57 ± 0.45	3.57 ± 0.48	3.57 ± 0.45	3.62 ± 0.45	0.829
MMSE	21.87 ± 2.40	22.00 ± 3.09	21.77 ± 2.31	21.81 ± 2.73	0.941
ADL	13.00 ± 0.01	13.13 ± 0.66	13.28 ± 1.82	13.00 ± 0.01	0.170

Note: Results are shown as *n* (%) for *χ*^2^ or Fisher’s exact test and mean ± SD for one-way ANOVA (two-tailed). *p* < 0.05 represents significant difference between groups. 1,25(OH)_2_VD_3_, 1,25-dihydroxy vitamin D_3_; 25(OH)VD_3_, 25-hydroxy vitamin D_3_; ADL, activities of daily living; BMI, body mass index; DHA, docosahexaenoic acid; HDL-C, high-density lipoprotein cholesterol; LDL-C, low-density lipoprotein cholesterol; MMSE, Mini-Mental State Examination; TC, total cholesterol; TG, triglyceride.

**Table 2:** Comparison of cognitive function scores at baseline and at 6-month and 12-month follow-ups.

		Intervention Time ^#^			Model 1		Model 2	
Cognitive Test	Groups	Baseline	6 Months	12 Months	*β* (95% CI)		*β* (95% CI)	
FIQ	VD_3_	91.91 ± 5.96	97.11 ± 3.92 ^ac^	99.40 ± 3.03 ^ac^	4.925 (3.316, 6.534) *	−2.818 (−4.428, −1.209) *	6.432 (4.822, 8.041) *	−6.271 (−7.880, −4.661) *
	DHA	93.36 ± 7.85	94.98 ± 3.84 ^ab^	95.79 ± 2.85 ^ab^	1.338 (−0.271, 2.948)	−6.405 (−8.014, −4.796) *	1.368 (−0.241, 2.977)	−11.334 (−12.944, −9.725) *
	VD_3_+DHA	93.67 ± 5.33	101.70 ± 4.02 ^abc^	107.44 ± 2.91 ^abc^	7.743 (6.134, 9.353) *	Ref	12.702 (11.093, 14.312) *	Ref
	Placebo	92.99 ± 5.51	93.28 ± 3.82	94.06 ± 3.13	Ref	−7.743 (−9.353, −6.134) *	Ref	−12.702 (−14.312, −11.093) *
VIQ	VD_3_	82.97 ± 3.64	89.34 ± 2.95 ^ac^	93.26 ± 3.47 ^ac^	6.305 (4.921, 7.689) *	−3.250 (−4.634, −1.866) *	9.792 (8.408, 11.176) *	−4.604 (−5.988, −3.220) *
	DHA	83.62 ± 6.96	85.46 ± 3.90 ^ab^	88.16 ± 3.80 ^ab^	1.757 (0.374, 3.141) *	−7.798 (−9.182, −6.414) *	4.034 (2.650, 5.418) *	−10.363 (−11.747, −8.979) *
	VD_3_+DHA	83.03 ± 3.77	92.67 ± 2.84 ^abc^	97.93 ± 2.14 ^abc^	9.555 (8.171, 10.939) *	Ref	14.397 (13.013, 15.781) *	Ref
	Placebo	83.43 ± 3.29	83.51 ± 3.72	83.93 ± 3.58	Ref	−9.555 (−10.939, −8.171) *	Ref	−14.397 (−15.781, −13.013) *
PIQ	VD_3_	104.54 ± 9.68	107.77 ± 7.15	107.73 ± 4.21	2.512 (−0.205, 5.228)	−2.064 (−4.781, 0.652)	1.460 (−1.257, 4.177)	−7.181 (−9.898, −4.464) *
	DHA	106.87 ± 11.28	108.12 ± 6.50	106.44 ± 4.46	0.531 (−2.186, 3.247)	−4.045 (−6.762, −1.329) *	−2.164 (−4.881, 0.552)	−10.805 (−13.522, −8.089) *
	VD_3_+DHA	108.22 ± 9.34	113.53 ± 7.76 ^abc^	118.62 ± 6.24 ^abc^	4.576 (1.859, 7.292) *	Ref	8.641 (5.925, 11.358) *	Ref
	Placebo	106.13 ± 9.04	106.87 ± 5.83	107.88 ± 5.21	Ref	−4.576 (−7.292, −1.859) *	Ref	−8.641 (−11.358, −5.925) *
Information	VD_3_	6.61 ± 1.04	7.12 ± 0.98 ^c^	7.36 ± 1.33 ^a^	0.445 (−0.059, 0.949)	−0.533 (−1.037, −0.029) *	0.678 (0.174, 1.182) *	−1.433 (−1.937, −0.929) *
	DHA	6.47 ± 1.08	6.40 ± 1.45 ^b^	7.22 ± 1.34 ^a^	−0.133 (−0.637, 0.371)	−1.111 (−1.615, −0.607) *	0.689 (0.185, 1.193) *	−1.422 (−1.926, −0.918) *
	VD_3_+DHA	6.36 ± 1.28	7.40 ± 1.26 ^ac^	8.53 ± 1.38 ^abc^	0.978 (0.474, 1.482) *	Ref	2.111 (1.607, 2.615) *	Ref
	Placebo	6.49 ± 1.64	6.56 ± 1.32	6.56 ± 1.32	Ref	−0.978 (−1.482, −0.474) *	Ref	−2.111 (−2.615, −1.607) *
Comprehension	VD_3_	3.72 ± 0.90	6.73 ± 0.90 ^ac^	8.72 ± 1.25 ^ac^	0.884 (0.330, 1.438) *	−0.427 (−0.981, 0.128)	1.204 (0.650, 1.758) *	−1.514 (−2.069, −0.960) *
	DHA	3.73 ± 1.87	4.67 ± 1.28 ^ab^	6.16 ± 1.27 ^ab^	0.113 (−0.441, 0.668)	−1.197 (−1.752, −0.643) *	0.256 (−0.298, 0.810)	−2.462 (−3.016, −1.908) *
	VD_3_+DHA	3.84 ± 1.18	7.83 ± 1.04 ^abc^	10.36 ± 0.95 ^abc^	1.311 (0.756, 1.865) *	Ref	2.718 (2.164, 3.273) *	Ref
	Placebo	4.16 ± 0.96	4.11 ± 1.23	4.11 ± 1.23	Ref	−1.311 (−1.865, −0.756) *	Ref	−2.718 (−3.273, −2.164) *
Arithmetic	VD_3_	6.13 ± 1.50	6.77 ± 1.71 ^a^	6.86 ± 1.73 ^a^	1.002 (0.345, 1.660) *	−1.009 (−1.666, −0.351) *	1.979 (1.322, 2.637) *	0.236 (−0.421, 0.894)
	DHA	6.40 ± 1.71	6.60 ± 1.53 ^a^	6.84 ± 1.63 ^a^	0.990 (0.332, 1.647) *	−1.021 (−1.679, −0.364) *	−0.300 (−0.957, 0.358)	−2.043 (−2.700, −1.385) *
	VD_3_+DHA	6.18 ± 1.19	7.07 ± 1.19 ^a^	7.59 ± 1.27 ^abc^	2.011 (1.353, 2.668) *	Ref	1.743 (1.085, 2.400) *	Ref
	Placebo	6.07 ± 1.40	6.02 ± 1.42	6.01 ± 1.43	Ref	−2.011 (−2.668, −1.353) *	Ref	−1.743 (−2.400, −1.085) *
Similarities	VD_3_	6.90 ± 1.69	7.90 ± 1.69 ^ac^	9.52 ± 1.88 ^a^	0.264 (−0.196, 0.724)	−0.058 (−0.518, 0.401)	0.176 (−0.284, 0.636)	−0.181 (−0.641, 0.279)
	DHA	7.54 ± 1.93	8.53 ± 1.93 ^ab^	7.89 ± 1.99 ^b^	−0.290 (−0.750, 0.170)	−0.612 (−1.072, −0.153) *	0.566 (0.107, 1.026) *	0.209 (−0.251, 0.669)
	VD_3_+DHA	6.98 ± 1.65	8.99 ± 1.65 ^ab^	9.37 ± 1.62 ^ac^	0.322 (−0.137, 0.782)	Ref	0.357 (−0.103, 0.817)	Ref
	Placebo	7.12 ± 1.65	7.12 ± 1.65	7.77 ± 1.87	Ref	−0.322 (−0.782, 0.137)	Ref	−0.357 (−0.817, 0.103)
Digit span	VD_3_	4.99 ± 0.69	5.81 ± 0.82 ^ac^	6.13 ± 1.17 ^ac^	3.056 (2.598, 3.515) *	−0.977 (−1.435, −0.519) *	5.045 (4.587, 5.503) *	−1.510 (−1.969, −1.052) *
	DHA	4.88 ± 2.50	4.93 ± 1.89 ^b^	5.08 ± 1.74 ^b^	0.978 (0.520, 1.436) *	−3.055 (−3.514, −2.597) *	2.467 (2.008, 2.925) *	−4.088 (−4.547, −3.630) *
	VD_3_+DHA	5.16 ± 0.92	6.41 ± 0.98 ^abc^	7.82 ± 0.99 ^abc^	4.033 (3.575, 4.492) *	Ref	6.555 (6.097, 7.014) *	Ref
	Placebo	5.09 ± 0.44	5.03 ± 1.70	5.03 ± 1.70	Ref	−4.033 (−4.492, −3.575) *	Ref	−6.555 (−7.014, −6.097) *
Vocabulary	VD_3_	7.60 ± 0.56	8.01 ± 0.77 ^c^	7.72 ± 1.12	0.677 (0.111, 1.242) *	−0.257 (−0.822, 0.309) *	0.777 (0.212, 1.342) *	−0.690 (−1.256, −0.125) *
	DHA	7.46 ± 2.09	7.31 ± 1.69 ^b^	7.97 ± 1.20 ^a^	0.244 (−0.321, 0.809)	−0.689 (−1.255, −0.124)	0.500 (−0.065, 1.065)	−0.968 (−1.533, −0.402) *
	VD_3_+DHA	7.52 ± 0.66	7.99 ± 0.69 ^c^	7.82 ± 0.94	0.933 (0.368, 1.499) *	Ref	1.467 (0.902, 2.033) *	Ref
	Placebo	7.51 ± 0.50	7.66 ± 1.32	7.46 ± 1.50	Ref	−0.933 (−1.499, −0.368) *	Ref	−1.467 (−2.033, −0.902) *
Digit symbol	VD_3_	9.72 ± 2.60	10.54 ± 3.16	8.73 ± 1.21 ^a^	0.146 (−0.633, 0.925)	−0.164 (−0.943, 0.615)	0.409 (−0.371, 1.188)	−0.495 (−1.274, 0.285)
	DHA	10.34 ± 3.45	10.98 ± 3.00	8.71 ± 1.15 ^a^	−0.040 (−0.819, 0.739)	−0.350 (−1.129, 0.429)	0.179 (−0.600, 0.958)	−0.724 (−1.503, 0.055)
	VD_3_+DHA	10.83 ± 2.99	12.16 ± 3.11 ^abc^	14.27 ± 2.81 ^abc^	0.310 (−0.469, 1.089)	Ref	0.903 (0.124, 1.682) *	Ref
	Placebo	10.44 ± 2.57	10.48 ± 2.60	10.49 ± 2.51	Ref	−0.310 (−1.089, 0.469)	Ref	−0.903 (−1.682, −0.124) *
Picture completion	VD_3_	6.52 ± 2.08	7.29 ± 2.14 ^a^	8.47 ± 1.83 ^ac^	0.794 (−0.243, 1.831)	−0.494 (−1.531, 0.542)	−1.030 (−2.066, 0.007)	−4.415 (−5.452, −3.379) *
	DHA	6.84 ± 1.90	7.01 ± 1.84	6.72 ± 2.02 ^b^	0.602 (−0.435, 1.639)	−0.687 (−1.723, 0.350)	−1.677 (−2.714, −0.641) *	−5.063 (−6.099, −4.026) *
	VD_3_+DHA	7.11 ± 1.54	8.11 ± 1.50 ^abc^	8.26 ± 2.00 ^ac^	1.288 (0.252, 2.325) *	Ref	3.386 (2.349, 4.422) *	Ref
	Placebo	6.62 ± 1.97	6.46 ± 2.00	6.74 ± 1.86	Ref	−1.288 (−2.325, −0.252) *	Ref	−3.386 (−4.422, −2.349) *
Block design	VD_3_	6.59 ± 2.32	6.61 ± 1.91	6.50 ± 2.01	0.931 (0.199, 1.664) *	−0.235 (−0.968, 0.497)	1.821 (1.088, 2.554) *	0.798 (0.065, 1.530) *
	DHA	6.67 ± 1.72	6.33 ± 2.01	6.56 ± 1.55	0.333 (−0.400, 1.065)	−0.834 (−1.567, −0.102) *	−0.245 (−0.977, 0.488)	−1.268 (−2.001, −0.535) *
	VD_3_+DHA	6.42 ± 1.54	7.17 ± 1.81 ^c^	6.92 ± 2.07	1.167 (0.434, 1.899) *	Ref	1.023 (0.291, 1.756) *	Ref
	Placebo	6.20 ± 1.94	6.53 ± 1.67	6.67 ± 1.81	Ref	−1.167 (−1.899, −0.434) *	Ref	−1.023 (−1.756, −0.291) *
Picture arrangement	VD_3_	7.91 ± 1.95	8.24 ± 1.97	8.68 ± 1.97	0.548 (−0.265, 1.361)	0.026 (−0.787, 0.839)	0.625 (−0.189, 1.438)	−1.062 (−1.875, −0.249) *
	DHA	8.36 ± 2.40	8.51 ± 2.06	8.90 ± 2.03	0.157 (−0.656, 0.970)	−0.365 (−1.178, 0.448)	0.611 (−0.202, 1.425)	−1.075 (−1.889, −0.262) *
	VD_3_+DHA	8.72 ± 2.10	9.23 ± 2.07 ^b^	10.00 ± 2.15 ^abc^	0.522 (−0.291, 1.335)	Ref	1.687 (0.873, 2.500) *	Ref
	Placebo	8.46 ± 2.00	8.66 ± 2.28	8.82 ± 2.00	Ref	−0.522 (−1.335, 0.291)	Ref	−1.687 (−2.500, −0.873) *
Object assembly	VD_3_	7.84 ± 2.09	8.60 ± 2.11	8.86 ± 2.22	−0.312 (−1.044, 0.420)	−0.723 (−1.456, 0.009)	−0.556 (−1.289, 0.176)	−0.590 (−1.323, 0.142)
	DHA	8.31 ± 2.29	8.68 ± 2.11	9.31 ± 1.91	−0.667 (−1.399, 0.065)	−1.078 (−1.810, −0.346) *	−0.578 (−1.310, 0.154)	−0.612 (−1.344, 0.120)
	VD_3_+DHA	8.50 ± 1.97	9.23 ± 2.15	10.58 ± 1.98 ^abc^	0.411 (−0.321, 1.143)	Ref	0.034 (−0.698, 0.766)	Ref
	Placebo	8.23 ± 1.87	8.44 ± 2.26	8.62 ± 2.11	Ref	−0.411 (−1.143, 0.321)	Ref	−0.034 (−0.766, 0.698)

Note: ^#^ Plus–minus values are mean ± SD for one-way ANOVA (two tailed); ^a^ *p* < 0.05 compared with the placebo group; ^b^ *p* < 0.05 compared with DHA group; ^c^ *p* < 0.05 compared with VD_3_ group. Results present estimates as *β* (95% CI) from linear mixed-effects models (LMMs); Model 1 and Model 2 *β* (95% CI) represent the overall treatment effect estimates at 6 and 12 months on cognitive tests, respectively. * *p* < 0.05 represents a significant interaction. All models were adjusted for age, sex, education levels, BMI, smoking, alcohol consumption, family history, and past history. FIQ, full intelligence quotient; PIQ, performance intelligence quotient; VIQ, verbal intelligence quotient.

**Table 3:** Comparison of blood biomarkers at baseline and at 6-month and 12-month follow-ups.

		Intervention Time ^#^			Model 1		Model 2	
Biomarkers	Groups	Baseline	6 Months	12 Months	*β* (95% CI)		*β* (95% CI)	
25(OH)VD_3_ (ng/mL)	VD_3_	19.97 ± 3.42	22.63 ± 2.73 ^ac^	25.74 ± 2.88 ^ac^	2.421 (1.165, 3.677) *	−0.688 (−1.944, 0.568)	5.706 (4.450, 6.962) *	−0.452 (−1.708, 0.804)
	DHA	19.76 ± 3.38	19.52 ± 3.65 ^b^	19.69 ± 3.84 ^b^	−0.472 (−1.728, 0.783)	−3.582 (−4.837, −2.326) *	−0.135 (−1.391, 1.120)	−6.293 (−7.549, −5.038) *
	VD_3_+DHA	19.54 ± 2.64	22.88 ± 3.36 ^ac^	25.77 ± 3.24 ^ac^	3.109 (1.853, 4.365) *	Ref	6.158 (4.902, 7.414) *	Ref
	Placebo	19.43 ± 3.38	19.67 ± 3.10	19.50 ± 2.86	Ref	−3.109 (−4.365, −1.853) *	Ref	−6.158 (−7.414, −4.902) *
1,25(OH)_2_VD_3_ (ng/mL)	VD_3_	31.10 ± 4.86	33.60 ± 5.30 ^ac^	36.20 ± 5.36 ^ac^	2.832 (0.853, 4.811) *	−0.985 (−2.964, 0.994)	5.187 (3.208, 7.165) *	0.227 (−1.752, 2.206)
	DHA	29.85 ± 4.34	30.18 ± 5.05 ^b^	29.93 ± 4.62 ^b^	0.660 (−1.318, 2.639)	−3.157 (−5.136, −1.178) *	0.161 (−1.818, 2.140)	−4.798 (−6.777, −2.820) *
	VD_3_+DHA	29.37 ± 5.14	32.86 ± 4.81 ^ac^	34.25 ± 4.56 ^ac^	3.817 (1.838, 5.796) *	Ref	4.959 (2.980, 6.938) *	Ref
	Placebo	30.21 ± 5.95	29.88 ± 5.36	30.13 ± 5.74	Ref	−3.817 (−5.796, −1.838) *	Ref	−4.959 (−6.938, −2.980) *
Erythrocyte DHA (ng/mL)	VD_3_	3.57 ± 0.48	3.61 ± 0.44 ^c^	3.54 ± 0.50 ^c^	0.008 (−0.102, 0.118)	−3.390 (−3.500, −3.281) *	−0.050 (−0.159, 0.060)	−3.773 (−3.882, −3.663) *
	DHA	3.57 ± 0.45	7.03 ± 0.96 ^ab^	7.41 ± 0.93 ^ab^	3.422 (3.312, 3.532) *	0.024 (−0.086, 0.134)	3.810 (3.700, 3.919) *	0.087 (−0.023, 0.196)
	VD_3_+DHA	3.62 ± 0.45	7.05 ± 1.01 ^ab^	7.37 ± 1.02 ^ab^	3.398 (3.288, 3.508) *	Ref	3.723 (3.613, 3.833) *	Ref
	Placebo	3.57 ± 0.45	3.60 ± 0.59	3.59 ± 0.60	Ref	−3.398 (−3.508, −3.288) *	Ref	−3.723 (−3.833, −3.613) *
Aβ_42/40_	VD_3_	15.59 ± 2.47	12.68 ± 2.71 ^a^	12.18 ± 3.05 ^a^	0.044 (−1.374, 1.463)	0.160 (−1.259, 1.578)	−0.623 (−2.041, 0.796)	0.057 (−1.361, 1.476)
	DHA	15.87 ± 2.49	15.81 ± 2.33	12.33 ± 2.81 ^a^	0.416 (−1.003, 1.834)	0.531 (−0.887, 1.949)	0.704 (−0.714, 2.123)	1.384 (−0.034, 2.803)
	VD_3_+DHA	15.97 ± 2.60	12.89 ± 2.36 ^ac^	8.89 ± 2.34 ^abc^	−0.115 (−1.534, 1.303)	Ref	−0.680 (−2.098, 0.739)	Ref
	Placebo	15.76 ± 2.24	15.82 ± 2.40	15.63 ± 2.37	Ref	0.115 (−1.303, 1.534)	Ref	0.680 (−0.739, 2.098)
APP	VD_3_	42.44 ± 19.61	42.92 ± 31.49	38.50 ± 24.96	0.494 (−10.000, 10.988)	3.659 (−6.835, 14.152)	−4.093 (−14.587, 6.400)	4.177 (−6.317, 14.671)
	DHA	44.92 ± 31.49	40.44 ± 19.61	40.89 ± 27.40	−4.473 (−14.967, 6.020)	−1.309 (−11.802, 9.184)	−4.189 (−14.682, 6.304)	4.081 (−6.412, 14.575)
	VD_3_+DHA	49.99 ± 35.51	46.83 ± 35.46	41.89 ± 33.01	−3.165 (−13.658, 7.329)	Ref	−8.270 (−18.764, 2.223)	Ref
	Placebo	42.91 ± 25.01	42.91 ± 25.01	43.07 ± 24.86	Ref	3.165 (−7.329, 13.658)	Ref	8.270 (−2.223, 18.764)
PS1	VD_3_	47.53 ± 36.70	42.42 ± 28.89	41.79 ± 27.81	−2.808 (−17.873, 12.257)	−0.075 (−15.140, 14.990)	−2.730 (−17.794, 12.335)	4.583 (−10.482, 19.649)
	DHA	49.98 ± 52.19	50.78 ± 56.10	51.40 ± 56.54	3.065 (−11.999, 18.129)	5.798 (−9.266, 20.862)	4.396 (−10.668, 19.460)	11.709 (−3.356, 26.773)
	VD_3_+DHA	47.34 ± 35.86	42.37 ± 35.80	37.10 ± 32.13	−2.733 (−17.797, 12.331)	Ref	−7.313 (−22.378, 7.751)	Ref
	Placebo	51.36 ± 40.21	49.11 ± 33.21	48.39 ± 32.60	Ref	2.733 (−12.331, 17.797)	Ref	7.313 (−7.751, 22.378)
PS2	VD_3_	10.43 ± 14.04	10.10 ± 14.02	11.16 ± 19.20	−0.410 (−5.753, 4.934)	−2.573 (−7.916, 2.771)	0.727 (−4.616, 6.070)	−1.184 (−6.527, 4.160)
	DHA	12.63 ± 21.42	12.95 ± 21.39	10.30 ± 15.14	0.249 (−5.094, 5.592)	−1.914 (−7.257, 3.429)	−2.320 (−7.664, 3.023)	−4.231 (−9.574, 1.112)
	VD_3_+DHA	10.36 ± 8.37	12.58 ± 6.79	12.24 ± 5.97	2.163 (−3.180, 7.506)	Ref	1.910 (−3.433, 7.254)	Ref
	Placebo	10.72 ± 9.74	10.79 ± 9.61	10.71 ± 9.62	Ref	−2.163 (−7.506, 3.180)	Ref	−1.910 (−7.254, 3.433)
IL-1β (pg/mL)	VD_3_	19.95 ± 3.79	18.21 ± 3.36 ^a^	15.90 ± 3.26 ^a^	−1.719 (−3.087, −0.351) *	0.959 (−0.410, 2.327)	−4.028 (−5.396, −2.659) *	5.535 (4.167, 6.903) *
	DHA	20.66 ± 3.47	18.40 ± 3.91 ^a^	16.71 ± 3.97 ^a^	−2.247 (−3.615, −0.879) *	0.431 (−0.937, 1.799)	−3.931 (−5.299, −2.563) *	1.507 (0.139, 2.876) *
	VD_3_+DHA	20.95 ± 3.52	18.24 ± 3.54 ^a^	15.38 ± 3.20 ^a^	−2.678 (−4.046, −1.310) *	Ref	−5.535 (−6.903, −4.167) *	Ref
	Placebo	20.84 ± 3.36	20.82 ± 3.38	20.82 ± 3.38	Ref	2.678 (1.310, 4.046) *	Ref	1.604 (0.236, 2.972) *
IL-18 (pg/mL)	VD_3_	30.08 ± 7.63	27.93 ± 7.92	27.43 ± 7.90	−1.626 (−4.272, 1.020)	1.178 (−1.468, 3.824)	−2.235 (−4.881, 0.411)	5.233 (2.588, 7.879) *
	DHA	29.69 ± 7.94	27.87 ± 7.67	28.37 ± 7.66	−1.306 (−3.952, 1.339)	1.498 (−1.148, 4.143)	−0.913 (−3.558, 1.733)	6.556 (3.910, 9.201) *
	VD_3_+DHA	30.06 ± 7.14	26.74 ± 7.03 ^a^	22.18 ± 6.89 ^abc^	−2.804 (−5.450, −0.158) *	Ref	−7.468 (−10.114, −4.823) *	Ref
	Placebo	30.50 ± 6.99	29.98 ± 7.15	30.09 ± 6.89	Ref	2.804 (0.158, 5.450) *	Ref	7.468 (4.823, 10.114) *
NLRP3	VD_3_	2.17 ± 4.78	2.00 ± 4.33	1.32 ± 1.44	−2.122 (−4.457, 0.213)	1.749 (−0.586, 4.084)	−3.632 (−5.967, −1.298) *	3.861 (1.526, 6.196) *
	DHA	1.68 ± 1.55	1.87 ± 3.51	2.15 ± 4.65	−1.875 (−4.210, 0.460)	1.996 (−0.339, 4.331)	−3.620 (−5.955, −1.286) *	3.873 (1.538, 6.208) *
	VD_3_+DHA	2.26 ± 4.80	1.91 ± 5.22	1.33 ± 2.45	−3.871 (−6.206, −1.536) *	Ref	−7.493 (−9.828, −5.159) *	Ref
	Placebo	1.78 ± 3.76	1.55 ± 2.51	1.54 ± 1.94	Ref	3.871 (1.536, 6.206) *	Ref	7.493 (5.159, 9.828) *
GSDMD	VD_3_	28.39 ± 5.28	26.27 ± 5.27	24.39 ± 5.80 ^a^	−3.024 (−3.719, −2.329) *	0.001 (−0.695, 0.697)	−3.544 (−4.239, −2.849) *	2.371 (1.676, 3.066) *
	DHA	27.80 ± 6.51	25.92 ± 6.53	23.81 ± 6.05 ^a^	−2.016 (−2.711, −1.321) *	1.008 (0.313, 1.703) *	−3.443 (−4.138, −2.748) *	2.473 (1.778, 3.168) *
	VD_3_+DHA	28.17 ± 5.35	24.30 ± 5.65 ^a^	20.30 ± 5.72 ^abc^	−3.024 (−3.719, −2.329) *	Ref	−5.915 (−6.610, −5.220) *	Ref
	Placebo	27.64 ± 6.99	27.64 ± 6.99	27.27 ± 6.30	Ref	3.024 (2.329, 3.719) *	Ref	5.915 (5.220, 6.610) *
Cleaved caspase-1	VD_3_	14.13 ± 2.00	11.13 ± 2.00 ^a^	10.58 ± 1.98 ^a^	−2.970 (−3.946, −1.994) *	0.175 (−0.801, 1.150)	−3.271 (−4.247, −2.295) *	3.682 (2.707, 4.658) *
	DHA	13.72 ± 1.86	11.73 ± 1.88 ^a^	10.28 ± 1.94 ^a^	−0.116 (−1.092, 0.860)	3.029 (2.053, 4.005) *	−3.410 (−4.386, −2.434) *	3.544 (2.568, 4.519) *
	VD_3_+DHA	13.73 ± 1.70	10.73 ± 1.70 ^ac^	7.81 ± 1.64 ^abc^	−3.145 (−4.121, −2.169) *	Ref	−6.954 (−7.929, −5.978) *	Ref
	Placebo	14.02 ± 1.94	14.04 ± 1.96	14.02 ± 2.03	Ref	3.145 (2.169, 4.121) *	Ref	6.954 (5.978, 7.929) *

Note: ^#^ Plus–minus values are mean±SD for one-way ANOVA (two tailed); ^a^ *p* < 0.05 compared with the placebo group; ^b^ *p* < 0.05 compared with the DHA group; ^c^ *p* < 0.05 compared with the VD_3_ group. Results present estimates as *β* (95% CI) from linear mixed-effects models (LMMs); Model 1 and Model 2 *β* (95% CI) represent the overall treatment effect estimates at 6 and 12 months on cognitive tests, respectively. * *p* < 0.05 represents a significant interaction. All models were adjusted for age, sex, education levels, BMI, smoking, alcohol consumption, family history, and past history. 1,25(OH)_2_VD_3_, 1,25-dihydroxy vitamin D_3_; 25(OH)VD_3_, 25-hydroxy vitamin D_3_; Aβ, amyloid beta; APP, Aβ protein precursor; Gasdermin D, GSDMD; PS, presenilin.

**Table 4:** Decomposition of total, direct, and indirect effects on cognitive function scores via pyroptotic pathways.

	*β* (95% CI)	SE_boot_	*p*	Effect Size
FIQ				
Total effects	11.075 (10.225, 11.790)	0.397	<0.001	
Direct effects	9.902 (9.048, 10.710)	0.438	<0.001	
Indirect effects	1.174 (0.612, 1.685)	0.276	<0.001	10.6%
IL-1β	0.152 (−0.024, 0.365)	0.100	0.128	1.4%
IL-18	0.165 (−0.074, 0.433)	0.131	0.208	1.5%
NLRP3	0.031 (−0.203, 0.267)	0.122	0.801	0.3%
GSDMD	0.856 (0.372, 1.349)	0.252	<0.001	7.7%
Cleaved caspase-1	0.274 (−0.159, 0.749)	0.225	0.222	2.5%
VIQ				
Total effects	9.501 (8.675, 10.385)	0.429	<0.001	
Direct effects	7.038 (5.927, 8.099)	0.56	<0.001	
Indirect effects	2.463 (1.577, 3.479)	0.48	<0.001	25.9%
IL-1β	0.235 (0.013, 0.564)	0.133	0.078	2.5%
IL-18	−0.013 (−0.345, 0.326)	0.169	0.939	−0.1%
NLRP3	0.201 (−0.067, 0.584)	0.162	0.059	3.5%
GSDMD	1.383 (0.692, 2.079)	0.361	<0.001	14.6%
Cleaved caspase-1	0.523 (−0.039, 1.103)	0.297	0.078	5.5%
PIQ				
Total effects	11.153 (9.748, 12.580)	0.699	<0.001	
Direct effects	10.753 (9.195, 12.209)	0.761	<0.001	
Indirect effects	0.400 (−0.328, 1.104)	0.370	0.280	3.6%
IL-1β	−0.043 (−0.342, 0.223)	0.142	0.761	−0.4%
IL-18	0.342 (0.018, 0.751)	0.185	0.065	3.1%
NLRP3	−0.289 (−0.637, 0.012)	0.162	0.075	−2.6%
GSDMD	0.101 (−0.577, 0.684)	0.305	0.739	0.9%
Cleaved caspase-1	−0.007 (−0.600, 0.593)	0.304	0.982	−0.1%

Note: Adjusting for age, sex, education levels, BMI, smoking, alcohol consumption, family history, and past history. FIQ, full intelligence quotient; Gasdermin D, GSDMD; PIQ, performance intelligence quotient; VIQ, verbal intelligence quotient.

## Data Availability

The original contributions presented in this study are included in the article/Supplementary Material. Further inquiries can be directed to the corresponding author.

## Supplementary Materials

The following supporting information can be downloaded at https://www.mdpi.com/article/10.3390/nu18172777/s1, File S1: CONSORT2026 Checklist; Table S1: Summary of model fit indices for the mediation models.

## Abbreviations

The following abbreviations are used in this manuscript:

1,25-dihydroxy vitamin D_3_	1,25(OH)_2_VD_3_
25-hydroxyvitamin D_3_	25(OH)VD_3_
Aβ	amyloid-β
AD	Alzheimer’s disease
ADL	activities of daily living
BMI	body mass index
CFI	Comparative Fit Index
DAMP	danger-associated molecular pattern
DHA	docosahexaenoic acid
DSM-IV	Diagnostic and Statistical Manual of Mental Disorders-IV
FIQ	full-scale intelligence quotient
HDL-C	high-density lipoprotein cholesterol
ITT	intention-to-treat
LDL-C	low-density lipoprotein cholesterol
MAR	missing at random
MCI	mild cognitive impairment
MMSE	Mini-Mental Status Examination
PIQ	performance intelligence quotient
RCT	randomized controlled trial
RMSEA	Root Mean Square Error of Approximation
SEM	structural equation modeling
SD	standard deviation
SRMR	Standardized Root Mean Square Residual
TC	total cholesterol
TG	triglyceride
TLI	Tucker–Lewis Index
Vitamin D_3_	VD_3_
VIQ	verbal intelligence quotient
WAIS-RC	Wechsler adult intelligence scale-revised in China
