# Supplementary material for: Effects of a Combined Vitamin D3 and Docosahexaenoic Acid Intervention on Cognitive Function and Blood Pyroptotic Biomarkers in Older Adults with Mild Cognitive Impairment: A 12-Month Randomized, Double-Blind, Placebo-Controlled Trial
Source: Nutrients. 2026 Aug 25;18(17):2777. doi: 10.3390/nu18172777 (PMC13567455; doi:10.3390/nu18172777)
Supplement: Supplementary file 1 [file nutrients-18-02777-s001.zip › Supplementary Table S1.pdf]

Table S1 Summary of model fit indices for the mediation models

	df	χ^2	CFI	RMSEA	TLI	SRMR	*P*
FIQ	40	108.668	0.964	0.069	0.922	0.024	<0.001
VIQ	40	131.911	0.947	0.08	0.884	0.026	<0.001
PIQ	40	94.174	0.971	0.061	0.936	0.023	<0.001

CFI, Comparative Fit Index; FIQ, full intelligence quotient; PIQ, performance intelligence quotient; RMSEA, Root Mean Square Error of Approximation; SRMR, Standardized Root Mean Square Residual; TLI, Tucker-Lewis Index; VIQ, verbal intelligence quotient.
